# Resolutions of Respect to Dr. Francis M. Odell

**Published:** 1904-01

**Authors:** 


### RESOLUTIONS OF RESPECT TO DR. FRANCIS M. ODELL.

The following resolutions were adopted at a regular meeting of the First District Dental Society of the State of New York, November 10, 1903:

Whereas, On October 11, 1903, Francis M. Odell, M.D., D.D.S., an honorary member of this Society and its secretary for some years in its early days, started on that unknown journey;

*Resolved,* That we, the members of the First District Dental Society, in regular session assembled, testify to the loss we feel in his departure from our midst;

To the loss the community has sustained since he has ceased to be able to give them the benefit of his well-trained mind and his skilful hand;

To his skill, which has preserved thousands of human teeth to remain a comfort to his former patients in their old age, which, in itself, is the highest praise we can bestow.

That we appreciate the work he has done for this Society and the scientific advancement of dentistry during his active career.

That the fortitude and patience he displayed during the last ten years of his life, constantly battling with a dread disease which not only prevented his practising his profession, but entailed untold suffering, makes his character stand out in a manner most creditable and worthy of emulation.

That we condole with his bereaved family; that a copy of these resolutions be sent to his widow, to the dental journals, and also be inscribed on our official minutes.

S. L. Goldsmith,
W. E. Hoag,
M. L. Rhein,
*Committee.*

